# Polysemous terms need context: A case of poorly differentiated spindle cell cutaneous squamous cell carcinoma

**DOI:** 10.1016/j.jdcr.2024.04.046

**Published:** 2024-05-21

**Authors:** Charlotte Read, Andrea Borba, Dan Lantz, Daniel Berg

**Affiliations:** a Department of Dermatology, University of Washington, Seattle, Washington; b Department of Medicine, Imperial College London, London, UK; c Department of Dermatology, Geisinger University, Danville, Pennsylvania; d Dermatopathology Northwest, Bellevue, Washington; e The Polyclinic Seattle, Mohs and Dermatology, Seattle, Washington

**Keywords:** cutaneous squamous cell carcinoma, dermatopathology, Mohs surgery, pathology report, patient anxiety, sarcomatoid, spindle cell

## Introduction

In pathology reports and in the cutaneous squamous cell carcinoma (cSCC) literature, the term "sarcomatoid" is sometimes used as a descriptive term in reference to poorly differentiated cSCC with spindle cell features that resemble a sarcoma in routine histologic sections.^1^ Confusion may arise because "sarcomatoid" is also used in reference to a rare biphasic tumor with mixed malignant squamous epithelial and malignant mesenchymal stromal elements (representing true sarcoma), which carries a much worse prognosis. Today, as part of a federal mandate from the 21st Century Cures Act Final Rule, patients are provided immediate electronic access to test results including pathology reports. This has resulted in increased worry for some patients as well as increased messaging between patients and their providers, oftentimes to alleviate this anxiety.^2^ Our case report highlights the importance of providing context with the polysemous term “sarcomatoid” to avoid confusion in our patients.

## Case report

**Fig 1:**
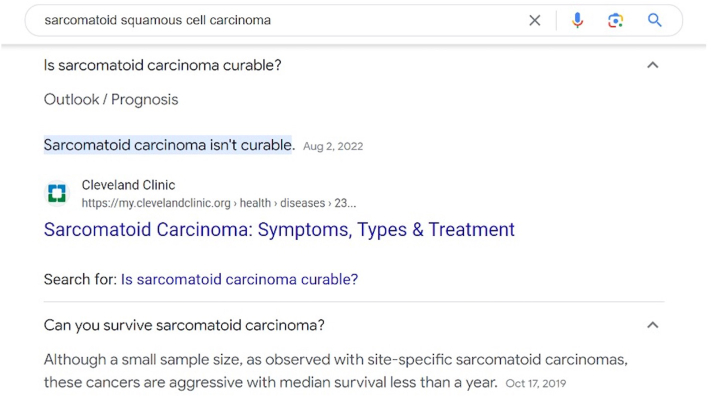
Google search results using the term “sarcomatoid squamous cell carcinoma” (accessed on 7/16/2023).

**Fig 2:**
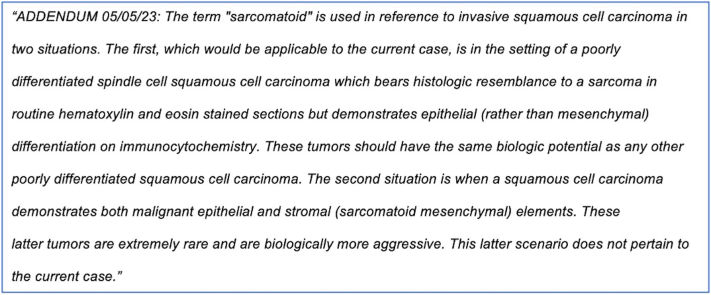
The addendum issued to the pathology report to explain the context for the term “sarcomatoid” in this patient’s case.

A 75-year-old male presented with a 1 cm papule on the right forehead, which was signed out as a “poorly differentiated sarcomatoid squamous cell carcinoma.” The tumor was composed of fascicles and sheets of poorly differentiated spindle cells that showed weak to moderate reactivity with pankeratin, partial reactivity with p63, and negative reactivity with S-100 protein on immunocytochemistry. Final staging was American Joint Commission on Cancer (AJCC) stage T1, and Brigham and Women’s Hospital (BWH) stage T2a based on poor differentiation. He underwent a magnetic resonance imaging of the head and neck, which showed a likely venous structure with an inability to exclude a possible perineural invasion at the supraorbital notch and a recommendation for further imaging later. Clear margins were obtained at Mohs surgery without evidence of microscopic perineural invasion, and the defect was primarily closed. Based on the poor differentiation, he chose to receive adjuvant radiation after surgery. Around 4 months postoperatively, the patient sent an urgent message after he conducted a Google search for “sarcomatoid squamous cell carcinoma” and was alarmed to find that his perceived diagnosis was associated with a median survival of 10 months (Fig 1). To alleviate his anxiety, his treating physician messaged him, and an addendum was issued to the pathology report (Fig 2).

## Discussion

Our case study highlights the confusion that can occur when patients access their own pathology reports, especially if more than one interpretation is possible. Specifically in this case, the use of the term "sarcomatoid" to describe a poorly differentiated spindle cell cSCC, despite being linguistically correct (the suffix “oid” means “resembling” or “like”), caused significant anxiety in a patient who mistook the use of this terminology to mean that he had a more aggressive tumor with elements of true sarcoma. To alleviate this concern, multiple additional communications were required between the dermatology provider and patient.

**Fig 3:**
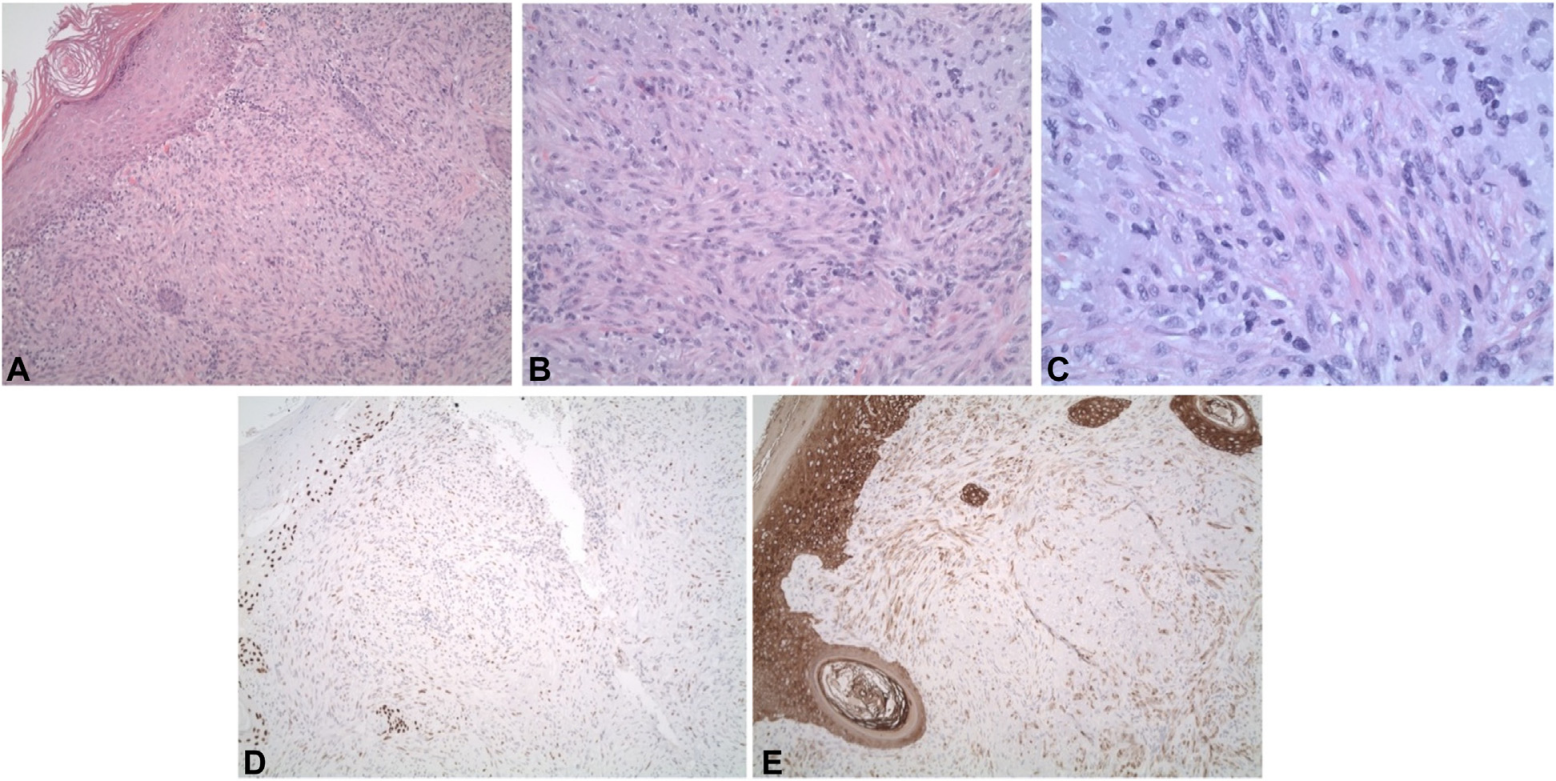
Histopathologic slides of this case demonstrating a cSCC with sarcomatoid features. **A,** Dermal proliferation of atypical spindle cells arranged in fascicles (H&E, ×100). **B,** Tumor cells have moderately pleomorphic, oval to spindled nuclei, vesicular nuclear chromatin, and indistinct eosinophilic cytoplasm (H&E, ×200). **C,** High-power view; typical and atypical mitoses present (H&E, ×400). **D,** Subtotal reactivity with pankeratin (IHC pankeratin, ×100). **E,** Subtotal reactivity with p63 (IHC p63, ×100). *cSCC*, Cutaneous squamous cell carcinoma; *H&E*, hematoxylin and eosin stain; *IHC*, immunohistochemistry.

The terms “sarcomatoid squamous cell carcinoma” or “sarcomatoid carcinoma” are commonly used in reference to cutaneous tumors in 2 primary scenarios: (1) as a descriptive term in the case of poorly differentiated cSCCs, which are predominately spindled, thus resembling a sarcoma by light microscopy, but which express epithelial markers by immunocytochemistry (such as in our patient, Fig 3); (2) in cases where the tumor is biphasic, exhibiting co-mingled epithelial and mesenchymal malignant elements.^3^ The prognosis in the first scenario is that of a poorly differentiated cSCC. The prognosis for the exceedingly rare and more aggressive second scenario is significantly worse and is generally considered to be poor regardless of the organ involved due to frequent metastasis and chemo-resistance.^4^ Such tumors have been variously termed sarcomatoid squamous cell carcinoma, carcinosarcoma, spindle cell carcinoma, and pleomorphic carcinoma.^5^

Our patient mistook his diagnosis of cSCC associated with sarcomatoid features to be a carcinosarcoma and, based on a Google search, inaccurately believed that his median survival was 10 months. With context provided in an addendum issued by the dermatopathologist, the treating provider was able to explain that the patient’s prognosis would be based on the overall stage of his poorly differentiated cSCC using the AJCC eighth edition or the BWH staging systems, which corresponded to the AJCC T1 stage or BWH T2a stage.

Following Mohs excision with free margins, the prognosis is usually good but can be poor if metastasis has occurred (which occurs in up to 5% of patients with cSCC).^6^, ^7^, ^8^ A recent review found that poor differentiation increased the relative risk of local recurrence by 2.4, the risk of nodal metastasis (NM) by 2.6, and the risk of disease-specific death (DSD) by 5.9.^9^ Patients with only one “high-risk” feature in the BWH classification are staged as T2a. The overall risk of NM and DSD for a BWH T2a cSCC is 5.2% and 1.2%, respectively. The addition of a second risk factor upstages a cSCC in BWH T2b. The overall risk of NM and DSD for a BWH T2b tumor is 24% and 17%, respectively.^10^

While the exact frequency of the term “sarcomatoid” to describe poor differentiation in cSCC with spindle cell features is unknown, in a search of reports from one of the author’s laboratories over the past 10 years, a diagnosis of sarcomatoid cSCC was rendered by different pathologists at least 40 times. This terminology is commonly used in the literature and is linguistically accurate. Nevertheless, to avoid confusion between patients and treating physicians, we would advocate limiting the use of “sarcomatoid.” Poorly differentiated cSCCs with spindle cell features can be reported as such, and rare truly biphasic tumors with elements of both squamous cell carcinoma and sarcoma may be best termed carcinosarcoma or biphasic carcinoma/sarcoma. If the term “sarcomatoid” is used to describe poor differentiation in cSCC with spindle cell features, an explanatory comment as part of the pathology report should be considered to explicitly state that this description does not imply the diagnosis of a true sarcoma. An explanatory comment to provide context and clarity may help to minimize patient anxiety and the subsequent additional provider burden that can occur with the immediate release of test results.

## Conflicts of interest

None disclosed.
